# Dental caries experience in first permanent molars and its associated factors among Iranian primary school-aged children: a cross-sectional study

**DOI:** 10.1186/s12903-026-09354-8

**Published:** 2026-08-08

**Authors:** Samaneh Shirahmadi, Salman Khazaei, Parisa Shokouhi, Shaghayegh Barkhordar, Saeed Bashirian, Amir Farhang Miresmaeili, Danial Aghabozorgi, Sima Kokabian

**Affiliations:** 1 Dental Research Center, Avicenna Institute of Clinical Sciences, Hamadan University of Medical Sciences, Hamadan, Iran; 2 Dental Implants Research Center, Avicenna Institute of Clinical Sciences, Hamadan University of Medical Sciences, Hamadan, Iran; 3 Oral Health Sciences Department, Faculty of Dentistry, University of British Columbia, Vancouver, BC Canada; 4 Autism Spectrum Disorders ResearchCenter, Institute of Neuroscience and Mental Health, Hamadan University of Medical Sciences, Hamadan, Iran; 5 Department of Dental, Sechenovskiy University of Medical Science, Moscow, Russia

**Keywords:** Dental Caries, Oral Health, Socioeconomic Factors, First Permanent Molar, Schoolchildren

## Abstract

**Background:**

Dental caries in first permanent molars is a highly prevalent condition in primary school-aged children and represents a substantial oral health burden in Iran. This study aimed to assess dental caries experience, based on the decayed, missing, and filled teeth index (DMFT) and its components in the first permanent molars, and to identify its associated factors among primary school-aged children in Hamadan, Iran.

**Methods:**

A cross-sectional study was conducted among 2,920 primary school students aged 7–12 years in Hamadan, Iran, between November 2019 and January 2020. Participants were selected through a three-stage cluster sampling method, in which schools and classes were randomly chosen, and all eligible students in the selected classes were included. Dental examinations were performed by a single calibrated dentist under natural light, using a World Health Organization (WHO) probe and disposable mirror, following the WHO criteria.

Data on students’ age, sex, residential area, history of dental pain in the past year, parental supervision, and oral health behaviors were collected using a structured interviewer-administered questionnaire, while information on parents’ education and occupation was obtained from school records. Data were analyzed using Stata version 14.0 (StataCorp LP). Caries experience in first permanent molars was defined as a binary outcome (DMFT > 0 vs. DMFT = 0), and associations with sociodemographic and oral health behavioral factors were examined using multiple logistic regression. A p-value < 0.05 was considered statistically significant.

**Results:**

The mean (SD) age of participants was 9.45 (1.71) years; 2,920 students were included (response rate 86.8%). The overall mean (SD) DMFT for first permanent molars was 1.56 (1.51), and the highest mean DMFT was observed at age 12 (mean 2.17, SD 1.50). The proportion of decayed first permanent molars decreased with age, from 90.44% at age 7 to 81.60% at age 12. In multiple logistic regression, where caries experience in first permanent molars was defined as DMFT > 0, age, sex, residential area, recent dental pain, and father’s education and occupation were independently associated with the odds of caries experience. The odds of caries experience increased with age (OR = 1.56, 95% CI [1.49–1.65], p< 0.001) and were higher in girls than in boys (OR = 1.42, 95% CI [1.21–1.67], p< 0.001). Children whose fathers had lower education had higher odds of caries experience (OR = 1.55, 95% CI [1.22–1.97], p < 0.001), and those living in lower socioeconomic residential areas also had increased odds (OR = 1.25, 95% CI [1.00–1.55], p = 0.04).

**Conclusion:**

The high proportion of decayed first permanent molars reflects a considerable burden of dental caries among primary school children in Hamadan. Higher DMFT was associated with age, female sex, and lower paternal education, underscoring the need for targeted preventive programs.

## Background

Permanent first molars (FPMs), which typically erupt between the ages of 6 and 7 years, are among the most critical teeth in the developing permanent dentition and play a central role in mastication, occlusion, and long-term oral function [1]. Due to their early eruption, complex occlusal morphology, deep pits and fissures, and posterior location in the oral cavity, these teeth are particularly susceptible to dental caries soon after eruption. Consequently, caries in FPMs is not only a common clinical finding in children but also serves as an indicator of early oral health vulnerability and gaps in preventive care [2–4]. Caries affecting these teeth may lead to pain, infection, premature tooth loss, malocclusion, impaired chewing and speech, and reduced quality of life [5–8].

Previous studies have shown that first molars are at high risk of caries during the years immediately following eruption [9]. Therefore, children aged 7 to 12 years represent a critical group for evaluating the status of these teeth, as this period encompasses both the eruption phase and the early post-eruptive years, during which FPMs are particularly vulnerable to caries progression and preventive interventions remain highly effective [10].

Studies conducted in various countries, including Poland, Brazil, Nepal, the United States, and Australia, have reported a substantial burden of caries in FPMs [11–18]. This burden is influenced by factors such as untreated caries in primary teeth, limited access to preventive services, and socioeconomic deprivation. However, many of these studies have primarily focused on estimating prevalence and have paid less attention to the determinants of caries experience within specific local contexts [14–18].

In Iran, dental caries remains one of the most significant public health challenges among children and adolescents [19–21]. According to the national oral health survey, the mean dmft in 5–6-year-old children was reported as 5.16, with 85.93% attributable to untreated decay. Among 12- and 13-year-old children, the mean DMFT values were 2.09 and 3.29, respectively, of which 81.83% and 86.89% were due to decay [20]. Although these national estimates provide important benchmarks for the primary and permanent dentitions, they do not report caries experience separately for first permanent molars, nor do they include specific estimates for 7–12-year-old primary school children.

Furthermore, Shoaee et al. reported that the DMFT index increased by more than 58% between 1990 and 2017, indicating a growing burden of caries in permanent teeth, including FPMs, nationwide [22]. In addition, a study using the Caries Assessment Spectrum and Treatment (CAST) index in 6–7-year-old children found that 25.30% to 31.20% of primary molars were affected by decay [23]. Despite the implementation of school-based oral health programs and preventive services in recent years, dental caries remains one of the most prevalent chronic diseases of childhood. FPMs are similarly affected, and available evidence suggests that these teeth are frequently involved during the primary school years [24–26].

Given that caries experience varies across populations depending on demographic and behavioral factors, up-to-date and locally representative data are essential for accurately describing disease distribution and identifying associated determinants. However, in Hamadan, there is limited recent and representative evidence regarding caries in permanent first molars among children aged 7 to 12 years. Existing local studies are often outdated and based on small or non-random samples, with insufficient reporting of data collection methods and examiner calibration [27, 28]. As a result, estimates of prevalence and identification of underlying factors remain uncertain, particularly for this critical age group, when first permanent molars are in their most vulnerable period. Moreover, the absence of an active annual oral health surveillance system has led to reliance on outdated data, making it difficult to monitor trends and prioritize local interventions effectively [24–26]. Therefore, conducting provincial studies with representative sampling and calibrated data collection is essential for a more accurate analysis of demographic and behavioral determinants. Because first permanent molars erupt earlier than other permanent teeth and are particularly vulnerable to caries, assessing them separately may provide information that is not fully captured by the overall DMFT index; therefore, tooth-specific analysis can help clarify the distribution and determinants of caries in this critical stage of dentition development [26, 29, 30].

Accordingly, the present study aimed to assess caries experience in permanent first molars among 7–12-year-old primary school children in Hamadan, Iran, and to identify associated demographic and behavioral factors. By focusing on a specific age group and a defined local population, this study provides context-specific evidence that may help clarify the distribution of FPM caries and support the development of early and targeted preventive interventions.

## Methods

### Design and calibration

This cross-sectional study was conducted between November 2019 and January 2020 in Hamadan, Iran, among 2,920 primary school students aged 7–12 years.

Oral examinations were performed according to World Health Organization (WHO) guidelines [31] by a single trained dentist who completed a formal training and clinical calibration program under the supervision of the Community Oral Health Department of Hamadan Dental School. Examiner reliability for the DMFT index was assessed in a calibration subsample. Inter-examiner agreement between the trained dentist and the supervising faculty member was quantified using the intraclass correlation coefficient (ICC), which was 0.86, indicating good reliability. In addition, intra-examiner reliability was assessed by repeating the examination in the same subsample after a short interval, and the ICC was 0.91, indicating excellent repeatability. To ensure data quality and minimize measurement bias, a standardized examination protocol was followed throughout the study, and fieldwork was periodically supervised to maintain consistency in examination and recording. The examiner was blinded to participants’ sociodemographic characteristics during the dental examination to reduce information bias.

### Sample size

The sample size was calculated using the following formula for estimating a mean: $\frac{{\left({z}_{1-\frac{\alpha}{2}}\right)}^{2}{\sigma}^{2}}{{d}^{2}}$  

where Z is the standard normal value corresponding to a 95% confidence level (1.96), σ is the standard deviation from previous studies 1.53 [32], and d is the desired precision of the estimate (0.076). The calculated base sample size was 1,556 students. After applying a design effect of 1.5 to account for cluster sampling, the sample size was adjusted to 2,334 students. To further accommodate a potential 20% rate of non-response and missing data, the final calculated sample size was 2,918 students. A total of 2,920 students completed both the dental examination and the questionnaire.

### Sampling method and outcome measures

A multistage cluster sampling design was used. In the first stage, the two educational districts in Hamadan (Districts 1 and 2) were included. Within each district, schools were stratified into three socioeconomic categories (high-, middle-, and low-income) based on a previously established municipal classification of school catchment areas in Hamadan, derived from a composite assessment of health-related, social-cultural, economic, infrastructural, and demographic indicators [33]. Schools were then proportionally selected from each socioeconomic stratum according to sex and student population.

**Fig. 1:**
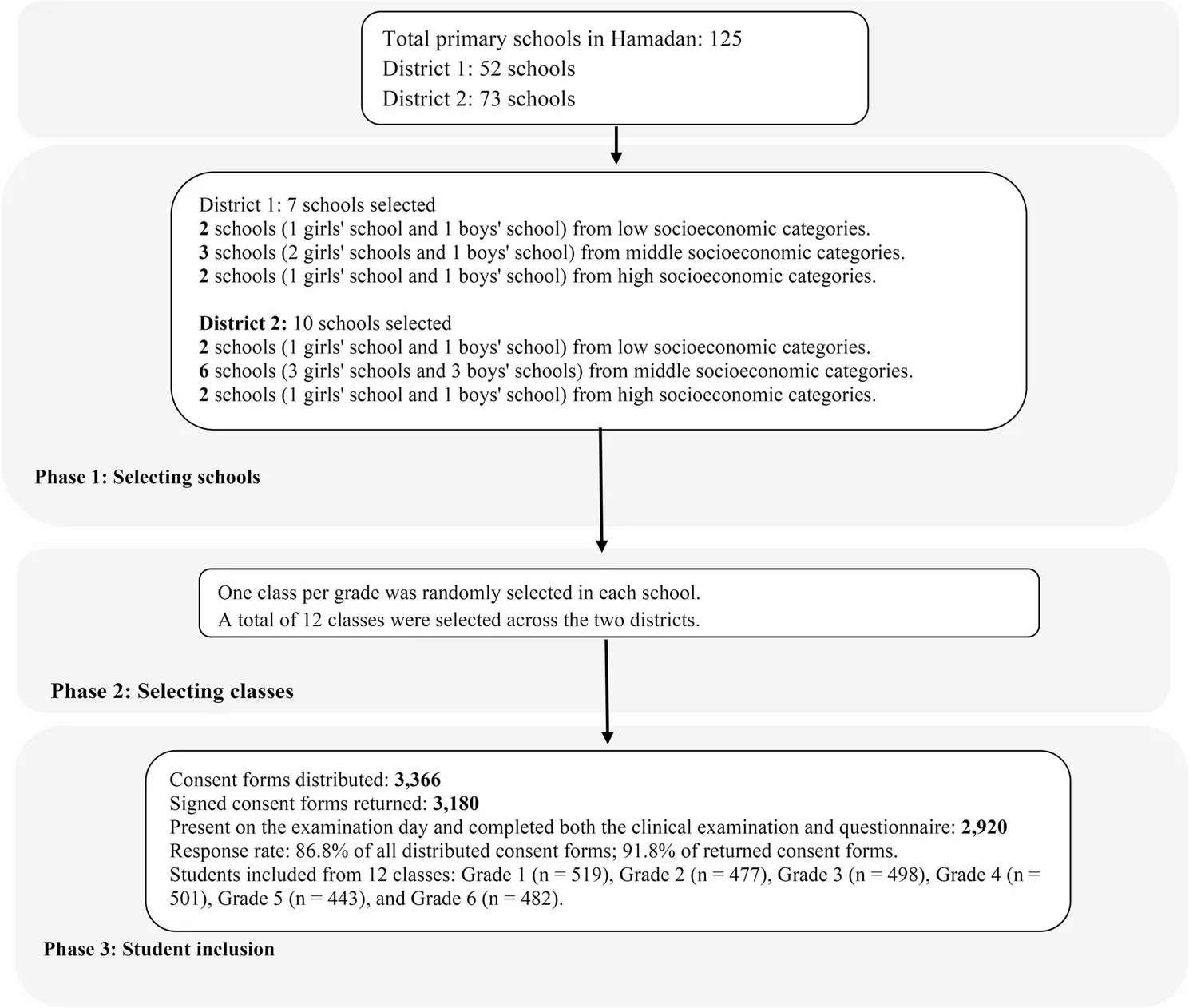
Flow diagram of multistage cluster sampling and participant inclusion

In the second stage, one class per grade was randomly selected in each chosen school. Written informed consent forms were distributed to all students in the selected classes during school hours, and parents/guardians were given one week to return the signed forms. A total of 3,366 consent forms were distributed, of which 3,180 were returned (94.5%). One week later, the research team returned to conduct on-site examinations in the classroom setting. Among students with returned consent forms, 2,920 were present on the examination day and completed both the clinical assessment and the interview-administered questionnaire, yielding an overall response rate of 86.8% (91.8% among those with returned consent forms). All eligible students who were present on the day of data collection, had a signed parent/guardian consent form, and personally agreed to participate were included; each underwent the clinical examination and completed the questionnaire on the same day (Fig. 1). This classroom-based, supervised data collection approach ensured complete participation among eligible students.

Children were excluded if they were absent on the day of examination, did not return a signed parental consent form, were outside the 7–12-year age range, had systemic disease, or were undergoing orthodontic treatment.

### Questionnaire

The questionnaire collected data on age, sex, dental pain experience, parental education, parental occupation, parental supervision, residential area, and tooth brushing behavior (frequency and duration). Tooth brushing frequency was categorized as never, rarely (twice a week), once daily, and twice or more times daily; duration was recorded as less than 2 min and 2 min or more. Data were collected using a structured interviewer-administered questionnaire.

Dental pain experience was assessed using a self-reported question: “In the last 12 months, how often have you had a toothache?” with response options of “never,” “seldom,” and “often.”

Parental education was categorized into three levels: less than high school diploma, high school diploma, and university degree. Parental occupation was categorized as Laborer, government employee, self-employed and unemployed/ housewife. Information on parental education and occupation was extracted from students’ school records.

Parental supervision of tooth brushing was assessed using a single question asking whether the child’s parents supervised their tooth brushing (yes/no).

Residential area (socioeconomic status) for each child was recorded as high-, middle-, or low-income, consistent with this classification.

The questionnaire was adapted from Bashirian et al. [32, 34], in which its validity and reliability were confirmed using confirmatory factor analysis (CFA). Further details on the development and validation process are provided in Bashirian et al. [34].

### Executive procedures

Following approval from the Department of Education, the necessary permissions for school visits were obtained. Schools were randomly selected, and visit schedules were coordinated with school principals and class teachers to facilitate questionnaire administration and oral examinations.

On the scheduled day, students were seated in a quiet classroom with adequate natural light, and the questionnaires were administered through face-to-face interviews. Dental examinations were performed to assess caries experience in the first permanent molars, rather than in the entire dentition. Examinations were conducted using a disposable dental mirror and a ball-ended WHO probe under natural light, with a portable examination light used when necessary.

Clinical findings were recorded by a trained clerk on WHO standard forms [35]. Examinations were carried out in empty classrooms, with students seated on high-back chairs and the examiner positioned in front of them. DMFT scores were determined by counting decayed, missing, and filled permanent teeth according to WHO criteria. No specific routine drying protocol was used before the caries examination; visible debris was removed only when necessary to ensure adequate visualization of the tooth surfaces.

### Statistical analysis

Descriptive statistics were calculated for DMFT and demographic variables. The mean numbers of decayed (DT), missing (MT), and filled (FT) teeth were calculated separately, and the relative contribution of each component was expressed as a percentage of the total DMFT score (DT + MT+ FT). For the main inferential analysis, the outcome variable was dichotomized as DMFT = 0 (caries-free) and DMFT ≥ 1 (caries experience).

Multiple logistic regression was used to assess demographic and behavioral predictors of any caries experience. This approach was adopted because the primary aim of the study was to identify factors associated with the presence of any caries experience in first permanent molars rather than to model the exact count of affected teeth. Given that 37.7% of children were caries-free and 62.3% had at least one affected tooth, and considering the highly skewed distribution of DMFT, binary logistic regression was considered the most appropriate and clinically interpretable method for the main analysis. Thus, the reported odds ratios (ORs) represent the odds of having at least one carious lesion in the first permanent molars (DMFT ≥ 1) compared with being caries-free (DMFT = 0).

All 2,920 participants were included in the final analysis, and no missing data were observed for the variables entered into the regression model. Complete participation was achieved through two follow-up visits in addition to the initial examination, which ensured that all selected students completed both the questionnaire and the clinical assessment.

Age and sex were included as independent variables along with other predictors. The “rare tooth brushing” category was merged with “never brushing,” as irregular brushing was not considered meaningfully different in terms of oral health behavior. Categories with very low frequencies were excluded to maintain model stability.

Given the multistage cluster sampling design, clustering at the school level was accounted for by estimating cluster-robust standard errors in all regression models. Because the sampling design was approximately self-weighting, with schools and classes selected with similar probabilities, sampling weights were not applied. Variables were entered into the multivariable logistic regression model based on theoretical relevance and prior evidence. Multicollinearity was assessed using variance inflation factors; the mean VIF was 1.28, with no value exceeding 2.5. Model fit was evaluated using the Hosmer–Lemeshow test, and discrimination was assessed using the area under the curve (AUC = 0.73). Adjusted odds ratios with 95% confidence intervals were reported, and *p* < 0.05 was considered statistically significant. All analyses were performed using Stata version 14.0 (StataCorp LP).

## Results

A total of 2,920 primary school children participated in the study, including 1,481 boys (50.7%) and 1,439 girls (49.3%), with ages ranging from 7 to 12 years. The overall mean (SD) DMFT was 1.56 (1.51), and 37.7% of participants were caries-free. The decayed (DT), missing (MT), and filled (FT) components accounted for 86.42%, 0.17%, and 13.41% of the total DMFT, respectively. The DT component accounted for 87.56% in boys and 86.00% in girls. By age, the D component accounted for 90.44% of DMFT in 7-year-olds and 81.60% in 12-year-olds. The highest mean (SD) DMFT by sex and age was observed in 12-year-old girls 2.23 (1.50). Among children with parental supervision, the M component was 0.07%, compared with 0.22% in those without parental supervision (Table 1).

**Table 1:** Mean DMFT of first permanent molars and distribution of DT, MT, and FT according to demographic variables

Variables	Categories	*N*	DMFT ^a^				Without caries ^b^ (%)
			Total Mean (SD)	DT%	MT%	FT%	
Total		2920	1.56 ± 1.51	86.42	0.17	13.41	37.7
gender	Boys	1481	1.44 ± 1.49	87.56	0.18	12.26	41.1
	Girls	1439	1.69 ± 1.52	86.00	0.16	14.00	34.2
Pvalue			< 0.001^c^	0.003^e^	0.96 ^e^	0.04 ^e^	< 0.001 ^e^
Age(year)	7	522	0.46 ± 0.90	90.44	0.00	10.66	73
	8	479	1.12 ± 1.31	93.50	0.00	6.50	46.8
	9	498	1.61 ± 1.44	88.70	0.00	11.30	31.9
	10	497	2.04 ± 1.51	86.88	0.19	12.93	23.7
	11	442	2.06 ± 1 0.55	85.46	0.54	14.00	25.3
	12	482	2.17 ± 1.50	81.60	0.09	18.31	22
*P*value			< 0.001^d^	< 0.001 ^e^	0.006 ^e^	< 0.001 ^e^	< 0.001 ^e^
Residential area	High income	720	1.53 ± 1.53	86.00	0.08	13.92	39.7
	Middle income	1359	1.52 ± 1.49	88.00	0.14	11.56	37.7
	Low income	841	1.63 ± 1.53	87.67	0.29	12.10	35.9
Pvalue			0.27 ^d^	0.04 ^e^	0.39 ^e^	0.01 ^e^	0.27 ^e^
Dental pain experience	Never	1223	1.48 ± 1.50	84.08	0.10	15.76	39.9
	Seldom	1133	1.65 ± 1.52	87.42	0.16	12.48	35
	Often	564	1.54 ± 1.54	90.68	0.33	9.00	38.3
Pvalue			0.02 ^d^	0.12 ^e^	0.40 ^e^	0.002 ^e^	0.02 ^e^
Father’s education	< High School diploma	1368	1.68 ± 1.51	91.13	0.34	8.53	34
	High School diploma	837	1.55 ± 1.52	86.00	0.00	14.00	38.4
	University degree	715	1.33 ± 1.46	77.00	0.00	23.00	43.9
Pvalue			< 0.001 ^d^	< 0.001 ^e^	0.01 ^e^	< 0.001 ^e^	< 0.001 ^e^
Mother’s education	< High School diploma	1290	1.71 ± 1.53	90.28	0.31	9.41	35.5
	High School diploma	1047	1.47 ± 1.52	84.00	0.06	15.94	40.8
	University degree	583	1.36 ± 1.43	82.00	0.00	18.00	41.5
Pvalue			< 0.001 ^d^	< 0.001 ^e^	0.04 ^e^	0.002 ^e^	< 0.001 ^e^
Father’s occupation	Laborer	588	1.85 ± 1.50	95.22	0.27	4.60	27.2
	Self-employment	1674	1.48 ± 1.51	85.56	0.16	14.28	40.6
	Government service	609	1.50 ± 1.50	78.79	0.10	21.89	39.6
	unemployed	49	1.42 ± 1.47	90.00	0.00	10.00	38.8
*P*value			< 0.001 ^d^	< 0.001 ^e^	0.64 ^e^	< 0.001 ^e^	< 0.001 ^e^
Mother’s occupation	Laborer	18	3.33 ± 1.23	100.00	0.00	0.00	5.6
	Self-employment	136	1.82 ± 1.52	82.00	0.00	18.00	30. 1
	Government service	189	1.41 ± 1.45	79.00	0.73	20.27	41. 8
	House wife	2577	1.54 ± 1.51	87.35	0.18	12.75	38
Pvalue			< 0.001 ^d^	0.002 ^e^	< 0.001 ^e^	0.004 ^e^	< 0.001 ^e^
Parental supervision	Yes	812	1.70 ± 1.50	86.28	0.07	13.65	39.8
	No	2108	1.50 ± 1.51	87.00	0.22	12.78	32.0.8
Pvalue			0.0001^c^	< 0.001 ^e^	0.33 ^e^	0.04 ^e^	< 0.001 ^e^

^a^DMFT of first permanent molars represents the number of decayed, missing, and filled first permanent molar teeth. DT, MT, and FT indicate the contribution of each component to the total DMFT of first permanent molars score and do not represent prevalence

^b^For the regression analyses, DMFT of first permanent molars was dichotomized as 0 = caries-free and ≥ 1 = caries experience

^c^The independent t-test was used to compare mean DMFT of first permanent molars between two groups

^d^One-way ANOVA was used to compare mean DMFT of first permanent molars among three or more groups

^e^The chi-square test was used to compare categorical variables, including caries-free status and the distributions of DT, MT, and FT components

At the tooth level, 10,804 first permanent molars were examined. The proportion of decayed teeth was higher in the mandible than in the maxilla (40.0%, 2172/5429 vs. 32.9%, 1772/5375; *p* < 0.001). Regarding oral health behaviors, 1,573 children (53.8%) reported never brushing or brushing rarely, and 1,514 (51.8%) brushed for less than 2 min per session (Table 2).

**Table 2:** Tooth-level caries status of first permanent molars and participants’ toothbrushing behavior

Tooth-level clinical findings	Totally	Maxillary	Mandibular	*P* value ^a^
Number of excluded teeth (partially erupted and unerupted)	876	465	411	-
Number of examined teeth	10,804	5375	5429	-
Decayed N (%) ^b^	3944 (36.5%)	1772 (32.9%)	2172 (40.0%)	Chi-squared = 859.79, *p* < 0.001
Missed N (%) ^b^	8 (0.07%)	3 (0.05%)	5 (0.09%)	Chi-squared = 0.005, *p* = 0.99
Filled N (%) ^b^	504 (4.6%)	228 (4.2%)	376 (6.9%)	Chi-squared = 737.23, *p* < 0.001
Sound N (%) ^b^	6248 (57.8%)	3372 (62.7%)	2876 (52.9%)	Chi-squared = 556.04, *p* < 0.001
Child-level behavioral characteristics	Categories	N (%)		
Tooth brushing frequency per day	Never	275 (9.4)	-	-
	Rarely (twice a week)	1298 (44.4)	-	-
	Once	845 (29.0)	-	-
	Twice or more	502 (17.2)	-	-
Brushing time ^c^	< 2 min	1514 (51.8)	-	-
	≥ 2 min	1131 (38.7)	-	-

^a^The chi-square test was used to compare the distributions of decayed, missing, filled, and sound teeth between the maxillary and mandibular arches

^b^Percentages of decayed, missing, filled, and sound teeth indicate the proportion of teeth in each category among all examined teeth; these are tooth-level measures and do not represent person-level prevalence

^c^Brushing time was recorded only among children who reported brushing; therefore, participants in the “never brush” category were not included in this variable

In the multiple logistic regression analysis, the outcome variable was caries experience (DMFT ≥ 1 vs. DMFT = 0). Age, sex, residential area, dental pain experience, father’s education, and father’s occupation were significantly associated with caries experience. Increasing age was associated with higher odds of caries experience (AOR = 1.56, 95% CI: 1.49–1.65, *p* < 0.001). Girls were more likely than boys to have caries experience (AOR = 1.42, 95% CI: 1.21–1.67, *p* < 0.001). Children who often experienced dental pain were more likely to have caries experience than those who had never experienced dental pain (AOR = 1.40, 95% CI: 1.11–1.73, *p* = 0.003). Compared with children from high-income areas, those from low-income areas showed greater odds of caries experience (AOR = 1.25, 95% CI: 1.00–1.55, *p* = 0.04), whereas the association for middle-income areas was not statistically significant (AOR = 1.12, 95% CI: 0.92–1.37, *p* = 0.23). Children whose fathers had less than a high-school diploma were more likely to have caries experience than those whose fathers had a university degree (AOR = 1.55, 95% CI: 1.22–1.97, *p* < 0.001). In contrast, having a self-employed father was associated with lower odds of caries experience compared with having a manual worker father (AOR = 0.61, 95% CI: 0.49–0.76, *p* < 0.001; Table 3). Brushing frequency and brushing duration were entered into the initial model but were not independently associated with caries experience after adjustment and were therefore excluded from the final model.

**Table 3:** Association of demographic factors with DMFT of first permanent molars in multivariable logistic regression analysis

Predictor Variables	DMFT	
	Adjusted OR (95% CI)	*P*_value_
Age (year)	1.56(1.49–1.65)	< 0.001
Gender	-	-
Boy (Reference category)	-	-
Girls	1.42(1.21–1.67)	< 0.001
Dental Pain Experience	-	-
Never (Reference category)	-	
Seldom	1.20(1.01–1.46)	0.04
Often	1.40(1.11–1.73)	0.003
Residential area		
High income (Reference category)	-	-
Middle income	1.12(0.92–1.37)	0.23
Low income	1.25(1.00-1.55)	0.04
Father’s Education		
University degree (Reference category)	1.55(1.22–1.97)	< 0.001
High School Diploma	1.23(0.96–1.56)	0.07
< High School Diploma		
Father’s Occupation		
Laborer (Reference category)		
Self-employment	0.61(0.49–0.76)	< 0.001
Government service	0.82(0.61–1.10)	0.20

The dependent variable was DMFT of first permanent molars, dichotomized as 0 = caries-free and 1 = caries experience

*CI* Confidence interval, *OR* Odds ratio

## Discussion

The first permanent molars (FPMs) are considered the cornerstone of the developing dentition because they play a pivotal role in establishing functional occlusion, supporting masticatory function, and maintaining vertical control of the occlusal plane [36–38]. The present study showed a substantial burden of caries in first permanent molars among primary school children, with untreated decay being the predominant component and caries experience being significantly associated with age, sex, dental pain, and socioeconomic indicators.Consistent with previous studies from different regions of Iran, approximately half of 12-year-old children were reported to have caries in their FPMs [24, 39, 40]. In contrast, substantially larger proportions of children in high-income countries remain caries-free at this age including Switzerland 72.7% [41], Germany 81.3% [42], Ireland 73% [43]. Moreover, untreated caries was highly prevalent in our sample, with 70.8% of 10-year-olds and 69.9% of 12-year-olds presenting with at least one untreated decayed tooth. This pattern is comparable to findings from several low- and middle-income settings [13, 44–46] and exceeds reports from countries such as China [11] and Saudi Arabia [8], highlighting a substantial unmet need for preventive and restorative dental care. Taken together, these comparisons suggest that the burden observed in our sample reflects not only disease occurrence, but also delayed detection and incomplete treatment coverage, likely influenced by structural and preventive differences across health systems.In many developed countries, comprehensive public dental care systems and school-based oral health programs—combining oral health education, routine examinations, and evidence-based preventive measures such as fluoride application—have contributed to substantial reductions in caries and oral health inequalities [47–51]. In Iran, the Oral Health Promotion Program was introduced in 2015; however, available evidence suggests that dental caries remains a major public health concern among primary school children [21] and that program implementation has primarily emphasized fluoride varnish, with relatively limited integration of structured oral health education [21]. Previous evaluations indicate that combining educational strategies with preventive interventions is more effective than single-component approaches for improving children’s oral health outcomes [52, 53]. Together, these findings suggest that differences in prevention coverage, care delivery, and school-based support may partly account for the contrast between our findings and those reported in higher-income settings.

The present study did not directly measure health-system characteristics such as service availability, accessibility, cost, or waiting times; therefore, any explanation involving system-level barriers remains speculative and should be interpreted cautiously. Nevertheless, existing literature suggests several plausible contributors, including limited access to public dental services, particularly in underserved areas; insufficient insurance coverage; and high out-of-pocket costs associated with private care, which may discourage service utilization [54, 55]. In addition, the composition of the oral health workforce—characterized by a strong reliance on dentists and limited availability of mid-level providers—may constrain the delivery of preventive services and follow-up care [56]. Furthermore, limited parental awareness of available services or of the importance of timely management of FPMs may also contribute to delayed care-seeking [24, 57]. These factors likely interact, reinforcing one another; however, because health-system and behavioral measures were not directly collected, these pathways remain speculative and require confirmation in future studies.

Oral health-promoting behaviors, particularly toothbrushing practices, were suboptimal in this study: only 17.2% of participants reported brushing at least twice daily, whereas 29.0% brushed once daily and 44.4% brushed rarely. This prevalence is lower than reported in many high-income settings—where twice-daily brushing often exceeds 60–70%—and is also below levels observed in some programs with structured oral health promotion [58]. In the multivariable regression model, brushing frequency and duration were not significantly associated with caries experience. This may indicate that caries experience is influenced more by qualitative aspects of toothbrushing, including correct technique (e.g., brush angle, applied force, and motion), effective plaque removal from all tooth surfaces, particularly occlusal and interproximal areas, and regular use of fluoride-containing toothpaste [59]. In addition, dietary behaviors—especially frequent consumption of free sugars and sugary snacks—may attenuate any protective effect of brushing and contribute to residual confounding in caries development [60]. Accordingly, the effect of brushing frequency should be interpreted in the context of the broader behavioral environment rather than as an isolated protective factor. Overall, these findings indicate that increasing toothbrushing frequency alone—without improving hygiene quality and addressing dietary behaviors—is unlikely to sufficiently reduce the burden of dental caries. Moreover, the absence of key behavioral and preventive variables—including dietary sugar intake, fluoride exposure, dental attendance patterns, and fissure sealant history—should be considered when interpreting the socioeconomic gradients observed in this study, as these unmeasured factors may have contributed to residual confounding.

Age, sex, dental pain, and several socioeconomic indicators were significantly associated with caries experience in first permanent molars (FPMs). The observed increase in mean DMFT with age likely reflects cumulative exposure to cariogenic factors and a longer duration of FPMs being at risk since eruption [61]. However, component analysis shows that across all age groups the largest contribution to DMFT derives from the D (decayed) component, while the F (filled) component does not increase substantially with age. This pattern indicates accumulation of untreated disease rather than a rise in restorative care and underscores the need to prioritize early preventive measures immediately after FPM eruption. Reporting caries experience by age enables identification of “critical time windows” for school-based and community preventive interventions (for example, targeted sealant and fluoride programs soon after eruption) and helps planners allocate resources to the most time-sensitive, high-impact actions [62].

Regarding sex, the results of this study showed that although the mean DMFT was higher in girls, the distribution of its components revealed a lower proportion of decayed (DT) teeth and a higher proportion of filled (FT) teeth compared to boys; these findings are consistent with similar studies [63–65]. This pattern may reflect earlier tooth eruption in girls, coupled with relatively greater access to or willingness to receive dental treatment. Therefore, relying solely on total DMFT may erroneously assume equivalent treatment needs across sexes; oral health programs should consider not only overall DMFT scores but also the DT/FT distribution within each group, and prioritize groups with a higher proportion of untreated decay (DT) for preventive interventions and improved access to care [63–65].

In this study, self-reported dental pain was entered as an independent predictor to examine the relationship between symptomatic disease and measured caries burden. We acknowledge that dental pain is primarily a consequence of untreated caries rather than a causal antecedent; in this cross-sectional design the association should be interpreted as an indicator of unmet treatment need and disease progression rather than causality. This interpretation is supported by findings from similar studies, which have demonstrated a moderate to strong correlation between history of dental pain and dental caries experience, particularly evident in populations with lower socioeconomic status and reduced access to dental care [66–68].The strong association between pain and higher caries experience indicates that a considerable portion of disease has progressed to symptomatic stages, highlighting gaps in timely access to care and the need to strengthen early detection, referral pathways, and provision of affordable services [66–68].

Socioeconomic patterns observed here align with theoretical frameworks and prior evidence: families with limited resources frequently prioritize immediate basic needs, which can reduce capacity for preventive behaviors and timely care-seeking [69]. Living in disadvantaged areas may both limit access to preventive and therapeutic services and expose children to social norms and behaviors associated with unhealthy diets and neglect of oral health, factors that can synergistically increase caries risk [70]. Conversely, higher parental education and more favorable occupational status are generally associated with greater health literacy, parental awareness, and better access to resources that facilitate preventive behaviors and timely use of dental services [71–73]. These results concur with studies reporting the protective role of higher parental education [74, 75], although some studies have reported weaker or inconsistent associations [72, 76, 77]. However, because intermediate socioeconomic mechanisms (for example, household diet, access to dental services, or parental health literacy) were not directly measured, these pathways should be interpreted cautiously and warrant confirmation in future research.

Consistent with previous studies [44, 78–80], mandibular FPMs showed higher caries experience than maxillary molars, likely due to earlier eruption and deeper pits and fissures that favor plaque accumulation [44, 78–80]. It may also reflect an interaction between anatomical susceptibility and the local plaque-retentive environment, which together increase vulnerability in mandibular FPMs. Similar findings have been reported in regional Iranian studies [81–83].

This study has several limitations that should be considered when interpreting the findings. First, the cross-sectional design precludes any inference about temporality or causality, and the observed associations should therefore be interpreted as correlational rather than causal. Second, selection bias may have occurred because only students who were present on the examination day and returned signed parental consent forms were included; consequently, children with different oral health profiles or family characteristics may have been underrepresented. Third, several behavioral variables were self-reported, including toothbrushing frequency and duration, parental supervision, and dental pain experience, which may have introduced information bias because of recall error or social desirability bias. Although the examinations were standardized and the examiner was calibrated, some degree of misclassification cannot be excluded.

In addition, despite adjustment for key sociodemographic factors, residual confounding remains possible because several established determinants of caries experience were not measured, including dietary habits and sugar intake, fluoride exposure, dental service utilization, and parental oral health knowledge. Socioeconomic status was also assessed using area of residence rather than individual-level indicators, which may not fully capture within-area heterogeneity. Finally, the use of the DMFT index provides a useful summary measure of caries experience, but it does not capture lesion severity, surface-level involvement, or detailed treatment needs. These limitations may partly explain the lack of association observed for some behavioral variables and should be considered when interpreting the results. Taken together, these limitations suggest that some associations may be underestimated or influenced by unmeasured factors, and future longitudinal studies with more comprehensive exposure assessment are warranted.

## Conclusion

Caries in first permanent molars remains a significant oral health concern among primary school children. The present analysis demonstrated that demographic and socio-economic factors—particularly age, sex, history of dental pain, father’s occupation and educational level, and place of residence—were significantly associated with caries experience in first permanent molars. These findings underscore the role of social determinants in shaping oral health outcomes in this population.

Based on these findings, targeted preventive strategies and early interventions are needed, particularly for the at-risk groups identified in this study. Policy-makers should strengthen preventive and educational components within existing child oral health programs, with a specific focus on first permanent molars and socially disadvantaged children.

## Data Availability

The author confirms that all data generated or analyzed during this study are included in this published article.

## Abbreviations

DMFT: Decayed Missing and Filled Teeth
WHO: World Health Organization
FPMs: First Permanent Molars
SD: Standard Deviation

## References

[1] Espinoza-Espinoza G, Corsini G, Rojas R, Mariño R, Zaror C. The cost-utility of school-based first permanent molar sealants programs: a Markov model. BMC Oral Health. 2019;19(1):1–11. 10.1186/s12903-019-0990-3 PMC6937712 31888582

[2] Songur F, Simsek Derelioglu S, Yilmaz S, Koşan Z. Assessing the impact of early childhood caries on the development of first permanent molar decays. Front public health. 2019;7:186. 10.3389/fpubh.2019.00186 PMC6629786 31338357

[3] King NM, Shaw L, Murray JJ. Caries susceptibility of permanent first and second molars in children aged 5–15 years. Commun Dent Oral Epidemiol. 1980;8(3):151–8. 10.1111/j.1600-0528.1980.tb01278.x 6936113

[4] Sánchez-Pérez L, Irigoyen-Camacho ME, Molina-Frechero N, Zepeda-Zepeda M. Fissure depth and caries incidence in first permanent molars: a five-year follow-up study in schoolchildren. Int J Environ Res Public Health. 2019;16(19):3550. 10.3390/ijerph16193550 PMC6801809 31547490

[5] Li F, Jiang P, Yu F, Li C, Wu S, Zou J, et al. Comparison between fissure sealant and fluoride varnish on caries prevention for first permanent molars: a systematic review and meta-analysis. Sci Rep. 2020;10(1):2578. 10.1038/s41598-020-59564-5 PMC7018844 32055001

[6] Llena C, Calabuig E, Sanz JL, Melo M. Risk factors associated with carious lesions in permanent first molars in children: a seven-year retrospective cohort study. Int J Environ Res Public Health. 2020;17(4):1421. 10.3390/ijerph17041421 PMC7068253 32098396

[7] Taylor GD, Pearce KF, Vernazza CR. Management of compromised first permanent molars in children: cross-sectional analysis of attitudes of UK general dental practitioners and specialists in paediatric dentistry. Int J Pediatr Dent. 2019;29(3):267–80. 10.1111/ipd.12469 30657228

[8] Al-Samadani KH, Ahmad MS. Prevalence of first permanent molar caries in and its relationship to the dental knowledge of 9–12-year olds from Jeddah, Kingdom of Saudi Arabia. International Scholarly Research Notices. 2012;2012. 10.5402/2012/391068 PMC3313570 22461990

[9] Aras A, Dogan MS. Caries prevalence and severity in immature permanent first molar teeth in Sanliurfa city, Turkey. J Dent Indones. 2020;27(1):13–6.

[10] Torres PJ, Phan HT, Bojorquez AK, Garcia-Godoy F, Pinzon LM. Minimally invasive techniques used for caries management in dentistry. A review. J Clin Pediatr Dentistry. 2021;45(4):224–32. 10.17796/1053-4625-45.4.2 34534308

[11] Liu M, Xu X, Song Q, Zhang H, Zhang F, Lai G. Caries prevalence of the first permanent molar and associated factors among second-grade students in Xiangyun of Yunnan, China: A cross-sectional study. Front Pead. 2022;10:946176. 10.3389/fped.2022.946176 PMC9558722 36245725

[12] Que L, Jia M, You Z, Jiang L-c, Yang C-g. Prevalence of dental caries in the first permanent molar and associated risk factors among sixth-grade students in São Tomé Island. BMC Oral Health. 2021;21(1):1–10. 10.1186/s12903-021-01846-z PMC8479893 34583665

[13] Stoica SN, Moraru SA, Nimigean VR, Nimigean V. Dental Caries in the First Permanent Molar during the Mixed Dentition Stage. Maedica. 2023;18(2):246. 10.26574/maedica.2023.18.2.246 PMC10427106 37588835

[14] Baginska J, Rodakowska E, Milewski R, Kierklo A. Dental caries in primary and permanent molars in 7-8-year-old schoolchildren evaluated with Caries Assessment Spectrum and Treatment (CAST) index. BMC Oral Health. 2014;14(1):74. 10.1186/1472-6831-14-74 PMC4074582 24952612

[15] Agrawal SK, Bhagat T, Shrestha A. Dental caries in permanent first molar and its association with carious primary second molar among 6–11-year‐old school children in Sunsari, Nepal. Int J Dent. 2023;2023(1):9192167. 10.1155/2023/9192167 PMC10049846 36998329

[16] Chaves JC, Santos TRd M, MdWSd, Alexandria A, Fidalgo TKS. Assessment of dental caries and intervention in the first permanent molars of Brazilian children. Pesquisa Brasileira em Odontopediatria e Clínica Integrada. 2021;21:e0010.

[17] Vélez-León E, Albaladejo A, Pinos-Narváez P, Cordero-López MA, Verdugo S, Melo M. Prevalence of first permanent molar caries: an observational study in Ecuadorian pediatric population. J Clin Pediatr Dentistry. 2025;49(4):154–61.

[18] Broadbent JM, Page LF, Thomson W, Poulton R. Permanent dentition caries through the first half of life. Br Dent J. 2013;215(7):E12–E. 10.1038/sj.bdj.2013.991 24113990

[19] Pournaghi-Azar F, Asl-Aminabadi N, Jamali Z, Azami A, Hazem K, Azami-Aghdash S, et al. Status of decayed, missing, filled teeth index among Iranian children and adults: A systematic review and meta-analysis. J Res Clin Med. 2018;6(2):55–66.

[20] Khoshnevisan M, Ghasemianpour M, Samadzadeh H, Baez R. Oral health status and healthcare system in IR Iran. J Contemp Med Sci. 2018;4(3):107–18.

[21] Shirahmadi S, Khazaei S, Meschi M, Miresmaeili AF, Barkhordar S, Heidari A, et al. Dental caries experience in primary school-age children following Students’ Oral Health Promotion Program, Iran. Int J Dental Hygiene. 2022;20(3):453–64. 10.1111/idh.12561 34714594

[22] Shoaee S, Masinaei M, Moghaddam SS, Sofi-Mahmudi A, Hessari H, Shamsoddin E, et al. National and subnational trend of dental caries of permanent teeth in Iran, 1990–2017. Int Dent J. 2024;74(1):129–37. 10.1016/j.identj.2023.07.012 PMC10829359 37574408

[23] Babaei A, Pakdaman A, Hessari H, Shamshiri AR. Oral health of 6–7 year-old children according to the Caries Assessment Spectrum and Treatment (CAST) index. BMC Oral Health. 2019;19(1):20. 10.1186/s12903-018-0709-x PMC6337759 30654779

[24] Khoramrooz M, Mirrezaie SM, Emamian MH, Golbabaei Pasandi H, Dadgari A, Hashemi H, et al. Economic inequalities in decayed, missing, and filled first permanent molars among 8–12 years old Iranian schoolchildren. BMC Oral Health. 2023;23(1):728. 10.1186/s12903-023-03471-4 PMC10559400 37805469

[25] Vejdani J, Amrollahi N, Amrollahi M, Peirowfeiz Z, Alinejad D. Parental awareness about the presence of permanent first molars and its relation to DMFT index in 7-9-year-old children. J Iran Dent Association. 2018;30(4):165–72.

[26] Kamiab N, Kamalabadi YM, Fathollahi MS. DMFT of the first permanent molars, dmft and related factors among all first-grade primary school students in Rafsanjan urban area. J Dent. 2021;22(2):109. 10.30476/DENTJODS.2020.85573.1136 PMC8206597 34150947

[27] Maasom T, Mojarad F, Akhtari K. Evaluation of first permanent molars DMFT in 12 years old children in Hamadan city (2005). 2007.

[28] Rafatjou R, Ahmadi B, Azizzadeh M. Evaluation of the Relationship of the Permanent First Molar Enamel Defects And Dental Caries With Delivery Type and Birth Weight In 6-to 12-Year-Old Children in Hamadan. Avicenna J Dent Res. 2018;10(4):120–4.

[29] Li Y, Wang W. Predicting caries in permanent teeth from caries in primary teeth: an eight-year cohort study. J Dent Res. 2002;81(8):561–6. 10.1177/154405910208100812 12147748

[30] Pontigo-Loyola AP, de Lourdes Márquez-Corona M, Minaya-Sánchez M, Lucas-Rincón SE, Casanova-Rosado JF, Robles-Minaya JL, et al. Correlation between the caries status of the first permanent molars and the overall DMFT Index: A cross-sectional study. Medicine. 2020;99(5):e19061. 10.1097/MD.0000000000019061 PMC7004719 32000455

[31] Petersen P, Baez R. World Health Organization Oral Health Surveys Basic Methods. WHO Library Cataloguing-in-Publication Data. 2013.

[32] Bashirian S, Shirahmadi S, Seyedzadeh-Sabounchi S, Soltanian AR, Karimi-Shahanjarini A, Vahdatinia F. Association of caries experience and dental plaque with sociodemographic characteristics in elementary school-aged children: a cross-sectional study. BMC Oral Health. 2018;18(1):7. 10.1186/s12903-017-0464-4 PMC5763616 29321029

[33] Esmaeilzadeh H, sR Y. Evaluating development levels and ranking of hamadan citys counties via topsis and kopras models. Journal of regional planning. 2017;7(25):15–28.

[34] Bashirian S, Seyedzadeh-Sabounchi S, Shirahmadi S, Karimi‐Shahanjarini A, Soltanian AR, Vahdatinia F. Predictors of oral health promotion behaviors among elementary school children: Examination of an extended social cognitive theory. Int J Pediatr Dent. 2021;31(2):191–203. 10.1111/ipd.12659 32339312

[35] Organization WH. Oral health surveys: basic methods. World Health Organization; 2013.

[36] Cai B, Zhao X-G. Molar and incisor positioning to achieve occlusal and esthetic objectives. Esthetics and Biomechanics in Orthodontics. WB Saunders; 2015;180–96.

[37] Taylor GD, Bulmer V. Advances in knowledge and practice benefiting the health and management of first permanent molars in children. Br Dent J. 2025;238(2):92–8. 10.1038/s41415-024-8012-5 PMC11761069 39856289

[38] Stoica SN, Nimigean V, Vîrlan MJR, Nimigean VR. The pathology of the first permanent molar during the mixed dentition stage. Appl Sci. 2022;13(1):483.

[39] Mahboobi Z, Pakdaman A, Yazdani R, Azadbakht L, Shamshiri AR, Babaei A. Caries incidence of the first permanent molars according to the Caries Assessment Spectrum and Treatment (CAST) index and its determinants in children: a cohort study. BMC Oral Health. 2021;21(1):259. 10.1186/s12903-021-01612-1 PMC8120821 33985489

[40] Poureslami P, Pouradeli S, Poureslami H, Shahrokhi E. Evaluation of health status of first permanent molar teeth among 12-year-old students in rural areas of south of Kerman, Iran, 2016. J Oral Health Oral Epidemiol. 2017;7(1):33–8.

[41] Grieshaber A, Waltimo T, Haschemi AA, Bornstein MM, Kulik EM. Dental caries and associated factors in 7-, 12‐and 15‐year‐old schoolchildren in the canton of Basel‐Landschaft, Switzerland: Changes in caries experience from 1992 to 2021. Int J Pediatr Dent. 2024;34(2):169–78. 10.1111/ipd.13122 37807838

[42] Al-Ani A, Takriti M, Schmoeckel J, Alkilzy M, Splieth C. National oral health survey on refugees in Germany 2016/2017: caries and subsequent complications. Clin Oral Invest. 2021;25:2399–405. 10.1007/s00784-020-03563-3 PMC7966123 33011846

[43] Sharma V, Crowe M, Cassetti O, Winning L, O’Sullivan A, O’Sullivan M. Dental caries in children in Ireland: A systematic review. Commun Dent Oral Epidemiol. 2024;52(1):24–38. 10.1111/cdoe.12897 37515429

[44] Que L, Jia M, You Z, Jiang L-c, Yang C-g. Prevalence of dental caries in the first permanent molar and associated risk factors among sixth-grade students in São Tomé Island. BMC Oral Health. 2021;21:1–10. 10.1186/s12903-021-01846-z PMC8479893 34583665

[45] Nayab H, Shinwari N, Alikozai N, Sekandari A, Sahebi S. The Prevalence Of Dental Caries In 1st Permanent Molar Teeth Among Teenage Boys And Girls At FMIC Hospital, Kabul, Afghanistan: An Observational Study. J Comm Med Pub Health Rep. 2024;5(07):1–7.

[46] Abuaffan AH, Hayder S, Hussen AA, Ibrahim TA. Prevalence of dental caries of the first permanent molars among 6–14 years old Sudanese children. Indian J Dent Educ. 2018;11(1):13–6.

[47] Winkelmann J, van Ginneken E, Gomez Rossi J. Oral health care in Europe: Financing, access and provision. Eur J Pub Health. 2022;32(Supplement3):ckac129. 35833482

[48] Bramantoro T, Santoso CMA, Hariyani N, Setyowati D, Zulfiana AA, Nor NAM, et al. Effectiveness of the school-based oral health promotion programmes from preschool to high school: A systematic review. PLoS ONE. 2021;16(8):e0256007. 10.1371/journal.pone.0256007 PMC8357156 34379685

[49] Priya PG, Asokan S, Janani R, Kandaswamy D. Effectiveness of school dental health education on the oral health status and knowledge of children: A systematic review. Indian J Dent Res. 2019;30(3):437. 10.4103/ijdr.IJDR_805_18 31397422

[50] Akera P, Kennedy SE, Lingam R, Obwolo MJ, Schutte AE, Richmond R. Effectiveness of primary school-based interventions in improving oral health of children in low-and middle-income countries: a systematic review and meta-analysis. BMC Oral Health. 2022;22(1):1–20. 10.1186/s12903-022-02291-2 PMC9245251 35768801

[51] Renson C. Changing patterns of dental caries: a survey of 20 countries. Ann Acad Med Singapore. 1986;15(3):284–98. 3777835

[52] Cakar T, Harrison-Barry L, Pukallus M, Kazoullis S, Seow W. Caries experience of children in primary schools with long‐term tooth brushing programs: A pilot Australian study. Int J Dental Hygiene. 2017. 10.1111/idh.12275 28345213

[53] Borges-Yáñez SA, Castrejón-Pérez RC, Camacho MEI. Effect of a School-Based Supervised Tooth Brushing Program In Mexico City: A Cluster Randomized Intervention. J Clin Pediatr Dentistry. 2017;41(3):204–13. 10.17796/1053-4628-41.3.204 28422600

[54] Rezaei S, Hajizadeh M, Irandoost SF, Salimi Y. Socioeconomic inequality in dental care utilization in Iran: a decomposition approach. Int J Equity Health. 2019;18:1–11. 10.1186/s12939-019-1072-5 PMC6805463 31640703

[55] Woldemichael A, Rezaei S, Kazemi Karyani A, Ebrahimi M, Soltani S, Aghaei A. The impact of out-of pocket payments of households for dental healthcare services on catastrophic healthcare expenditure in Iran. BMC Public Health. 2021;21(1):1–8. 10.1186/s12889-021-11209-6 PMC8320192 34320939

[56] Bayat F, Vehkalahti MM, Akbarzadeh A, Monajemi F. Varying manpower alters dental health in a developing health care system. Int Dent J. 2022;72(3):360–5. 10.1016/j.identj.2021.04.003 PMC9275189 34134900

[57] Heydari A, Shahrabi M, Shafizadeh M, Anaraki EA, Aref M. Parental knowledge and awareness of the first permanent molar. Int J Clin Pediatr dentistry. 2018;11(5):382. 10.5005/jp-journals-10005-1544 PMC6379531 30787550

[58] Llodra JC, Phantumvanit P, Bourgeois DM, Horn V. LLL 2: an international global level questionnaire on toothbrushing and use of fluoride toothpaste. Int Dent J. 2014;64:20–6. 10.1111/idj.12128 PMC9376510 25209647

[59] Marsh P. Contemporary perspective on plaque control. Br Dent J. 2012;212(12):601–6. 10.1038/sj.bdj.2012.524 22722123

[60] Moynihan P, Kelly S. Effect on caries of restricting sugars intake: systematic review to inform WHO guidelines. J Dent Res. 2014;93(1):8–18. 10.1177/0022034513508954 PMC3872848 24323509

[61] Al-Samadani KH, Ahmad MS. Prevalence of first permanent molar caries in and its relationship to the dental knowledge of 9–12-year olds from Jeddah, Kingdom of Saudi Arabia. Int Sch Res Notices. 2012;2012(1):391068. 10.5402/2012/391068 PMC3313570 22461990

[62] Cooper AM, O’Malley LA, Elison SN, Armstrong R, Burnside G, Adair P et al. Primary school-based behavioural interventions for preventing caries. Cochrane Database Syst Reviews. 2013(5). 10.1002/14651858.CD009378.pub2 PMC11873670 23728691

[63] Beltrán-Aquilar E, Espinoza L, Li C-H, Lin M, Thornton-Evans G, Wei L. Oral Health Surveillance Report: Dental Caries, Tooth Retention, and Edentulism: 2017-March 2020. 2024.

[64] Kockanat A, Acipinar S. Evaluation of oral and dental health of institutionalized orphan children residing in children’s homes in Türkiye. BMC Oral Health. 2025;25(1):1829. 10.1186/s12903-025-07240-3 PMC12642361 41286857

[65] Villavicencio-Caparo E, Alvarracin-Duran AJ. Association between Body Mass Index and Decayed, Missing, and Filled Teeth (DMFT) in 12-year-old School Children. Open Dentistry J. 2025;19(1):e18742106364538.

[66] Slade GD. Epidemiology of dental pain and dental caries among children and adolescents. Community Dent Health. 2001;18(4):219–27. 11789699

[67] Kumar Y, Acharya S, Pentapati K. Prevalence of dental pain and its relationship to caries experience in school children of Udupi district. Eur Archives Pediatr Dentistry. 2014;15(6):371–5. 10.1007/s40368-014-0124-1 24683004

[68] Bakar A, Ningrum V, Lee A. Structural equation modelling of the complex relationship between toothache and its associated factors among Indonesian children. Sci Rep. 2020;10:13567. 10.1038/s41598-020-70104-z PMC7419561 32782302

[69] Council O. Policy on social determinants of children’s oral health and health disparities. Oral health. 2017.

[70] Oliveira RCd, Shafik S, Khalid H, Stellrecht E, McKernan SC. Multilevel Determinants of Adolescent Dental Care Access: A Scoping Review Using a Health Disparities Framework. INQUIRY: J Health Care Organ Provis Financing. 2026;63:00469580261427669. 10.1177/00469580261427669 PMC13009631 41832770

[71] Llena C, Calabuig E. Risk factors associated with new caries lesions in permanent first molars in children: a 5-year historical cohort follow-up study. Clin Oral Invest. 2018;22:1579–86. 10.1007/s00784-017-2253-5 29063383

[72] Peres M, Sheiham A, Liu P, Demarco FF, Silva AE, Assunção MC, et al. Sugar consumption and changes in dental caries from childhood to adolescence. J Dent Res. 2016;95(4):388–94. 10.1177/0022034515625907 26758380

[73] Mansur EK, Rothen ML, Gao SS. Impact of parental education and socio-economic status on children’s oral health behaviors. Frontiers Media SA; 2025. p. 1755103. 10.3389/froh.2025.1755103 PMC12745420 41473868

[74] Almajed OS, Aljouie AA, Alharbi MS, Alsulaimi LM, Aljouie A, ALHARBI M et al. The impact of socioeconomic factors on pediatric oral health: a review. Cureus. 2024;16(2):e53567. 10.7759/cureus.53567 PMC10914081 38445162

[75] Moghaddam LF, Vettore MV, Bayani A, Bayat A-H, Ahounbar E, Hemmat M, et al. The Association of Oral Health Status, demographic characteristics and socioeconomic determinants with Oral health-related quality of life among children: a systematic review and Meta-analysis. BMC Pediatr. 2020;20(1):489. 10.1186/s12887-020-02371-8 PMC7579886 33092562

[76] Veiga N, Pereira C, Amaral O. Prevalence and determinants of dental caries in Portuguese children. Procedia-Social Behav Sci. 2015;171:995–1002.

[77] Pawellek M, Kopf FM, Egger N, Dresch C, Matterne U, Brandstetter S. Pathways linking parental health literacy with health behaviours directed at the child: a scoping review. Health Promot Int. 2022;37(2):daab154. 10.1093/heapro/daab154 34668013

[78] Pontigo-Loyola AP, de Lourdes Márquez-Corona M, Minaya-Sánchez M, Lucas-Rincón SE, Casanova-Rosado JF, Robles-Minaya JL et al. Correlation between the caries status of the first permanent molars and the overall DMFT Index: A cross-sectional study. Medicine. 2020;99(5):e19061. 10.1097/MD.0000000000019061 PMC7004719 32000455

[79] Mubaraki S, AlOlyan R, AlBrekeit J, AlFouzan S, Abosharkh M, AlSaeri N et al. Prevalence of caries in first permanent molar among children in Saudi Arabia: a retrospective study. Eur Rev Med Pharmacol Sci. 2022;26(20):7550–55. 10.26355/eurrev_202210_30026 36314345

[80] Almugla YM. Prevalence of missing first permanent molars in a selected population in a university dental clinic setting: a retrospective radiographic study. Int J Clin Pediatr dentistry. 2021;14(2):269. 10.5005/jp-journals-10005-1941 PMC8343692 34413605

[81] Sadeghi M. Prevalence and bilateral occurrence of first permanent molar caries in 12-year-old students. J Dent Res Dent Clin Dent prospects. 2007;1(2):86. 10.5681/joddd.2007.015 PMC3525931 23277840

[82] Rezaie M, Ghapanchi J, Haghnegahdar A, Khojastehpour L, Khorshidi H, Heidari H. A radiographic evaluation of missing of permanent first molars in a group of Iranian children and adults: a retrospective study. Int J Dent. 2018;2018(1):5253965. 10.1155/2018/5253965 PMC5902067 29808093

[83] AGHA HF, Enshaei M. DMFT evaluation of Upper and Lower First Permanent Molars in Patients examined in Oral Medicine &Diagnosis Department at Faculty of Dentistry, Tehran University of Medical Sciences (1997). 2002.

